# The hydraulic resistance paradox in rapid narrow pipe waterflow

**DOI:** 10.1038/s41598-020-78109-4

**Published:** 2020-12-09

**Authors:** Richard Dvorský, Ladislav Svoboda, Jiří Bednář

**Affiliations:** 1 Nanotechnology Centre, VŠB-Technical University of Ostrava, 17. listopadu 15/2172, 708 33 Ostrava, Czech Republic; 2 IT4Innovations National Supercomputing Center, VŠB-Technical University of Ostrava, 17. listopadu 15/2172, 708 33 Ostrava, Czech Republic

**Keywords:** Physics, Fluid dynamics

## Abstract

In this work we present experimental results of cross-sectional speed of water flow in narrow cylindrical metal tubes at high pressure gradients up to 1.1 GPa$\cdot$m^−1^. The measurement draws attention to the paradoxical behaviour of flowing water in internal diameters less than 250 $\upmu$m. At constant pressure gradient, its cross-section speed decreases with decreasing diameter in accordance with the classical hydrodynamic prediction for turbulent flow in rough cylindrical tube. However for very low diameters below 250 $\upmu$m, the cross-section speed rises again and reaches almost the maximum theoretical value of the outflow speed for the appropriate pressure without energy loss caused by contraction or hydraulic friction. Our contribution describes mainly experimental character of the new phenomenon and its motivation is to promptly provide the material for further study to the professional public.

## Introduction

At present, the issue of fluid flow in very narrow pipes becomes more acute. The Steinke and Kandlikar^1^ overview summarizes the results of 35 papers, which focused on the study of the flow in this special field. However only in Adams et al.^2^, the Reynolds number reached the maximum order of 10^4^ (Re $\approx$ 21 429), which is comparable to maximum values of Re $\approx$ 28 500 in our work. But the diameter of the pipe was more than 1 mm and the paper also does not contain explicit values of flow speed. In our previous work^3^, we observed a significant deviation from the predictions of classical hydrodynamics for turbulent flow during technical use of extreme hydraulic impacts in fast moving water (v > approx. 50 ms^−1^) in cylindrical metallic pipe. Based on it we conducted a new experiment, which systematically mapped the dependence of the cross-sectional speed of water flow on the inside diameter *d* of the tube in the region (0.13 $\le$ d $\le$ 0.50) mm. These experimental results are presented in this brief statement.

## Materials and methods

**Figure 1:**
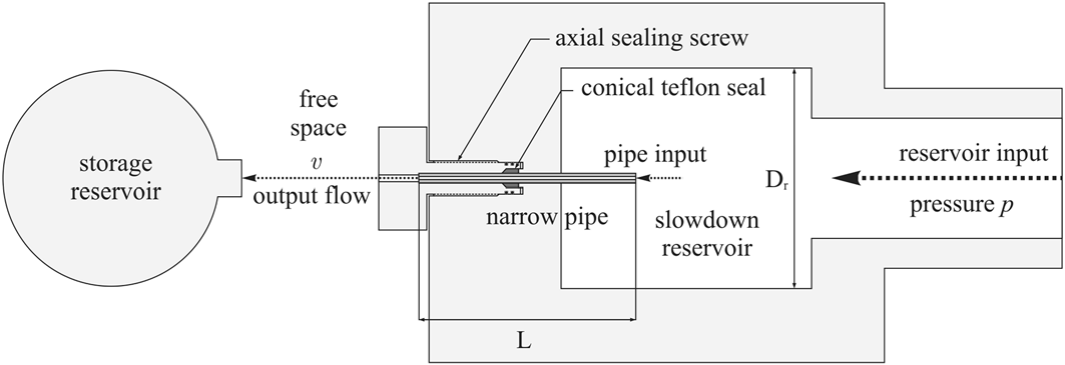
Scheme of the experiment. The pipe is placed in the middle of the slowdown reservoir and sealed by axial screw with teflon conical seal. We then observed the dependence of the cross-section speed *v* in narrow pipe on the pressure *p* of the fluid in reservoir.

For this flow rate analysis, we used narrow stainless pipe (VICI AG International and Poppe + Potthoff GmbH) of length L = 20 mm with metallographically perpendicular ends. High-pressure water was generated in a Krenzle 24.060 high-speed plunger pump and pressure pulses were eliminated to values below 0.3 % by a high-pressure damper and a high-pressure tank with a volume of 1000 ml. To avoid unwanted speed affection of the fluid at the narrow pipe input, we designed a slowdown reservoir with negligible cross-sectional speed $v\cdot \left( d/D_{r}\right) ^{2}\rightarrow 0$. The pipe input was then placed in the middle of slowdown reservoir with diameter D_r_ = 40  mm and the pipe was fixed by axial sealing screw (see Fig. 1). The real cross-section speed was determined by simple calculation of $v=4Q/\pi d^{2}t$ from the integral measurement of the total volume of water *Q* leaked from a tube of diameter *d* to the storage reservoir at the given time *t*. Prior to experiment, we flushed the device with high-pressure deionized water, which was degassed in vacuum under ultrasound. The experiment was then performed with conventional deionized water.

## Results and discussion

**Figure 2:**
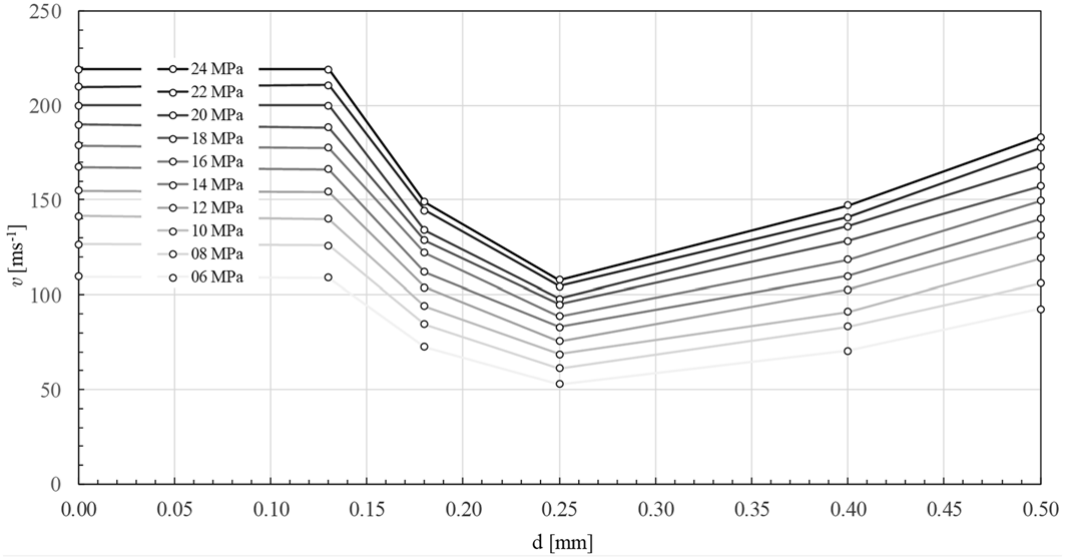
Dependence of the mean cross-sectional flow speed *v* in metal tubes of length 20 mm with internal diameter *d* for input pressure p = (6, 8, 10, 12, 14, 16, 18, 20, 22, 24) MPa from values in Table 1.

Experimental results of this measurement for the different inner diameters *d* of the tube are shown in Table 1, that contains five columns of measured cross section speed $v\left( p\right)$ with respect to the input pressure *p* and the inner diameters *d* = 0.13, 0.25, 0.40 and 0.50 mm (also graphically displayed in Fig. 2) and the last column represents the maximum theoretical outflow speeds $v_{theor}$ for the respective pressures without energy losses caused by contraction and hydraulic friction.

**Table 1:** Experimental results of cross-section speed *v* for given input pressures *p* with maximum uncertainty u_v_ = 2.0 ms^−1^ and their comparison to theoretical values $v_{theor}$ without energy losses caused by contraction and friction.

$\upsilon$ [$\hbox {ms}^{{1}}$]	*d* [mm]	*d* [mm]	*d* [mm]	*d* [mm]	*d* [mm]	${\varvec{\upsilon }}$_theor._
*p* [MPa]	0.50	0.40	0.25	0.18	0.13	
**6**	92.52	70.44	52.75	72.72	109.04	109.54
**8**	106.01	83.44	61.25	84.58	126.01	126.49
**10**	119.25	91.11	68.79	94.13	139.89	141.42
**12**	131.18	102.55	75.53	103.84	154.20	154.92
**14**	139.98	109.82	83.00	111.85	166.19	167.33
**16**	149.61	118.46	88.57	122.10	177.70	178.89
**18**	157.42	128.46	95.07	128.64	188.35	189.74
**20**	167.77	135.97	98.04	134.14	200.00	200.00
**22**	177.54	140.91	104.37	144.51	210.65	209.76
**24**	183.19	147.20	107.76	149.06	219.13	219.09

The pressure loss $\Delta p$ in water flow with density $\rho$ and cross sectional speed *v* in tube with diameter *d* and length *L* is described by well-known Darcy–Weissbach equation^4^

1 $$\begin{aligned} \Delta p=\lambda \cdot \frac{L}{d}\cdot \frac{1}{2}\rho {{v}^{2}}, \end{aligned}$$

where $\lambda$ is a frictional loss coefficient. In order to express the frictional loss coefficient $\lambda$, we have to formulate a series of equations with respect to the nature of the flow, which is described by Reynolds number and relative roughness. The analysis of experimental data in Table 1 shows that the studied flow is characterized by Reynolds number Re $\gg$ 10^4^, which corresponds to the quadratic area with intense turbulence.

**Figure 3:**
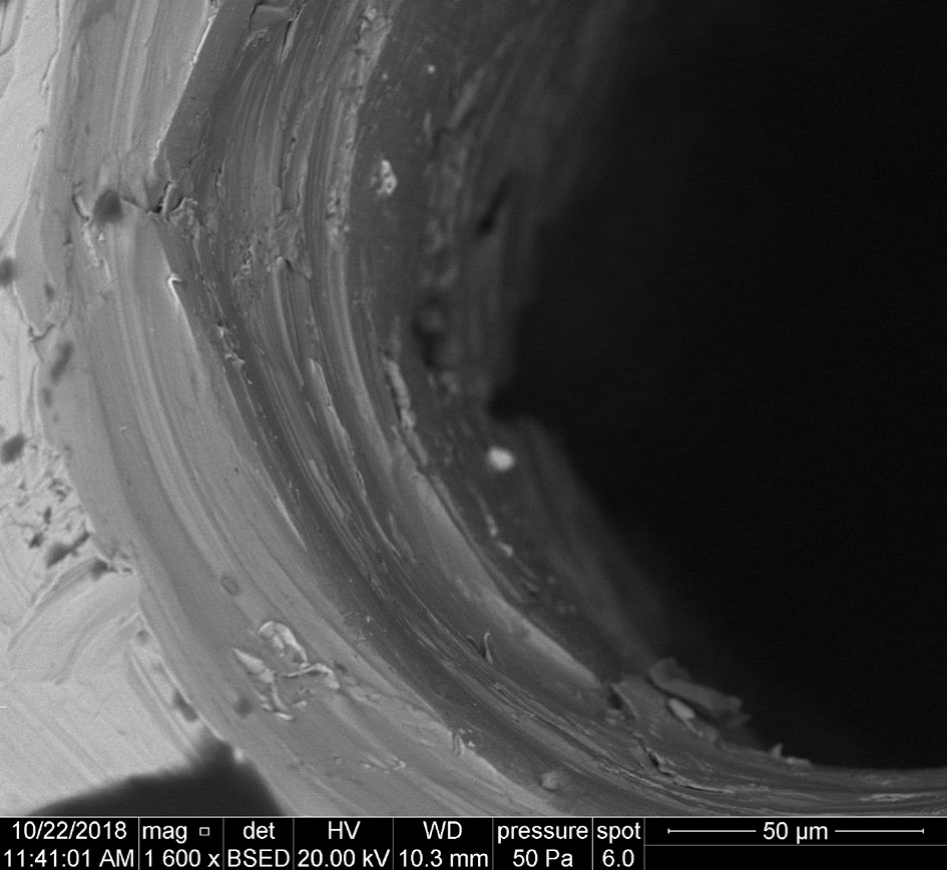
Scanning electron micrograph of the tube’s inner wall. The relative roughness was analysed sectional by Mitutoyo Surftest SJ-401 with the resulting values within the range of 2–4 %.

The hydraulic flow losses of water with the above parameters fit the best into quadratic area of intense turbulence, which is described by Von Karman - Nikuradse loss coefficient^5,6^

2 $$\begin{aligned} \lambda ={{\left( 2\log \frac{d}{2\delta }+1.14 \right) }^{-2}}, \end{aligned}$$

that depends only on the relative roughness of the tube’s wall $d/2\delta$ (see Fig. 3). After substitution of Von Karman - Nikuradse formula of the loss coefficient (2) into Darcy–Weissbach equation (1), the value of the mean cross sectional flow speed *v* can be expressed in the form

3 $$\begin{aligned} \Delta p={{\left( 2\log \frac{d}{2\delta }+1.14 \right) }^{-2}} \frac{L}{d} \frac{1}{2}\rho {{v}^{2}}\rightarrow v=\sqrt{\frac{2d\Delta p}{\rho L}}\left( 2\log \frac{d}{2\delta }+1.14 \right) . \end{aligned}$$

Assuming a zero pressure at the outflow of the tube into the empty space and neglecting the influence of contraction at the inlet, the pressure loss $\Delta p$ is equal to the inlet pressure *p* at the tube’s orifice and the final speed formula for both parameters takes a form

4 $$\begin{aligned} v\left( p,d \right) =\sqrt{\frac{2dp}{\rho L}}\left( 2\log \frac{d}{2\delta }+1.14 \right) . \end{aligned}$$

**Figure 4:**
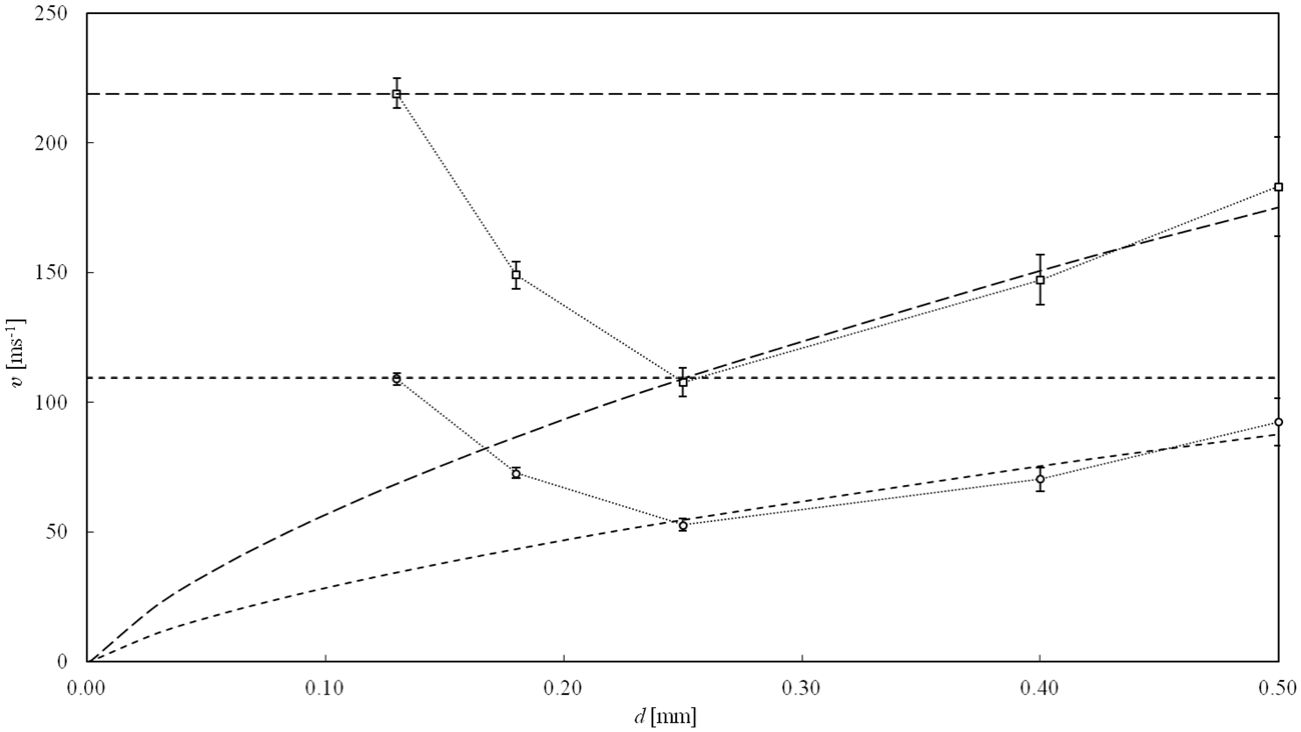
Experimentally determined dependence of the mean cross-sectional flow speed in the tube with length 20 mm on internal diameter *d* for inlet pressures 24 MPa (square points) and 6 MPa (circle points), which can be compared with theoretical curves according to (4), where the long and short dashed lines stands for 24 MPa and 6 MPa respectively. The corresponding horizontal lines represent the maximum theoretical values of the outflow speed without energy losses caused by contraction and friction for the inlet pressures. The dotted connectors for both set of data are inserted only to indicate the affinity of points for a given pressure value and does not represent the true dependence between them.

At the end in the Fig. 4 we directly compared experimental data for pressures 6 MPa and 24 MPa from Table 1 with predicted theoretical dependencies $v\left( d\right)$ from equation (4). We can clearly see a significant deviation at very small diameters < 250 $\mu$m for all inlet pressure values. For tubes with diameter in the area around 130 $\mu$m, the outflow speed is then getting closer to its maximum theoretical values without energy losses caused by contraction and hydraulic friction.

Turbulent entry length L_h_ = 10d reaches values in the range of 1.3–5 mm^7^. Due to a much larger length of the pipe L = 20 mm and the extreme nature of the flow, it is very problematic consider the entry length effect as a realistic parameter in the possible explanation of this deviation from the theoretical extrapolation (4). One of the possibilities to explain this paradoxical behaviour is a surface cavitation on the interface between flowing water and strongly curved wall of the tube. Such flow mode would describe an idealized model of the collective movement of the water column, separated from the wall by a cavitation layer of gas and vapour. It is generally known that cavitation occures at the sharp edge of nozzle inlet^8^. At the inlet of the tube (Fig. 3) is a smooth metallographic cut perpendicular to its axis, which forms a sharp edge around the inner circumference. The beginning of the flow forms just behind the sharp edge in the region of low contraction a very thin layer of surface cavitation, which then fully surrounds the fluid flow^9^. At high pressure gradients, the cavitation envelope stretches very quickly in the direction of flow, and in the order of tens of $\mu$s reaches the level of the tube outlet^10^. After reaching the level of the tube outlet, the opened cavitation layer is filled with air and the hydraulic flip phenomenon occurs^9–12^. The hydraulic flip has been experimentally studied in several publications, namely Cui et al.^11^ and Sou et al.^12^. Cui et.al performed experiments with a maximum nozzle length of 4 mm and minimum diameter of 0.8 mm, which gives the maximum ratio of L/d = 3.2. Sou et al. had a maximum nozzle length of 10 mm and minimum diameter of 0.5 mm, which gives the maximum ratio of L/d = 20. For the contribution of our paper, we consider experimental findings that the hydraulic flip occurs even at very high ratios of length to diameter L/d = 154, which significantly exceed the values in the experiments performed so far. If the column of fluid moves in the axial direction without contact with the tube wall, the question is, how can the fluid preserve its radial stability in the expected turbulence, especially regarding turbulent velocity fluctuations in individual microvolumes. Based on Reynolds decomposition, the instantaneous velocity ${\mathbf {u}}\left( {\mathbf {x}},t \right)$ in the vicinity of **x** can be expressed as the sum of temporal mean ${{\left\langle {\mathbf {u}}\left( {\mathbf {x}},t \right) \right\rangle }_{t}}$ and fluctuation component $\mathbf {{u}'}\left( {\mathbf {x}},t \right)$

5 $$\begin{aligned} {\mathbf {u}}\left( {\mathbf {x}},t \right) ={{\left\langle {\mathbf {u}}\left( {\mathbf {x}},t \right) \right\rangle }_{t}}+\mathbf {{u}'}\left( {\mathbf {x}},t \right) ,\text { }{{\left\langle \mathbf {{u}'}\left( {\mathbf {x}},t \right) \right\rangle }_{t}}=0. \end{aligned}$$

Due to above Reynolds decomposition, the total kinetic energy of the fluid in the column of volume *V* can be expressed as the sum of the kinetic energies of the collective axial translation $E_{k}^{\text {tr}}$ and the internal turbulent fluctuations $E_{k}^{\text {fl}}$

6 $$\begin{aligned} {{E}_{k}}\left( V \right) =E_{k}^{\text {tr}}\left( V \right) +E_{k}^{\text {fl}}\left( V \right) . \end{aligned}$$

According to Table 1 and Fig. 2, experimentally determined cross-section outlet speeds from a tube with a diameter of 0.13 mm are within the measurement uncertainty $u_v$ equal to the maximum theoretical flow rate at the pressure *p* in the reservoir

7 $$\begin{aligned} v\approx {{v}_{theor.}}=\sqrt{\frac{2p}{\rho }} . \end{aligned}$$

The experimental value of cross-section speed *v* is then completely equivalent to the energy of collective axial translation $E_{k}^{\text {tr}}$ of the fluid column and the necessary pressure energy for the energetic excitation of the internal turbulent fluctuations $E_{k}^{\text {fl}}$ is completely missing

8 $$\begin{aligned} {{E}_{k}}\left( V \right) \approx E_{k}^{\text {tr}}\left( V \right) . \end{aligned}$$

**Figure 5:**
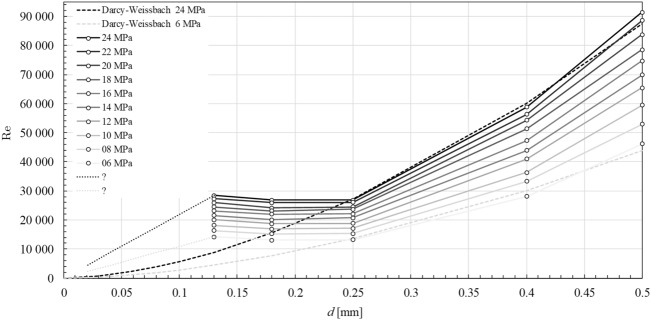
Experimental points $\circ$ of dependence of the Reynolds number Re in metal tubes of length 20 mm on internal diameter d for input pressure p = (6, 8, 10, 12, 14, 16, 18, 20, 22, 24) MPa from values in Table 1. Above 0.25 mm in diameter, the experimental values for 6 MPa and 24 MPa are in good agreement with the theoretical prediction of the Darcy–Weissbach equation with Von Karman–Nikuradse loss coefficient (dashed lines). In possible extrapolation below 0.13 mm, we can guess the flow rate has already reached a constant theoretical maximum and the Reynolds number would only be a linear function of the diameter d (dotted lines).

The logical conclusion is therefore absence of turbulent velocity fluctuations in the column of leaking fluid. In this situation, we could consider describing the flow inside the column by a model of the laminar velocity profile in the slip flow mode. However, the maximum value in the tube axis profile must correspond to the theoretical flow rate, so the only acceptable model approximation seems to be a “perfect slip”, which corresponds to a constant speed profile in the “piston flow” mode. Dependences of the Reynolds numbers on the pipe diameter (Fig. 5) have approximately constant course within the measurement uncertainty in the range of 0.25–0.13 mm diameter. With decreasing diameter, the flow rate approaches the “piston flow” mode and reaches its maximum around 0.13 mm (see Fig. 4). In acceptable approximation, the radial stability of the fluid column is ensured by a constant velocity profile without turbulent fluctuations and viscous friction. The cohesive pressure of water at very small column diameters, which can compensate the dynamics of possible radial pressure fluctuations, can also be considered as an additional stabilizing effect. The cohesive pressure was calculated to be about 1.1 kPa based on the Laplace-Young equation for the column diameter d = 0.13 mm. The real application of the mentioned stabilization effect is highly debatable for lower flow rates, but with further reduction of the column diameter below 0.13 mm, an increase in significance can be expected. Authors are aware that the paper does not provide a completely exact quantitative description of the flow mechanism, but our main intention is to acquaint the wider hydraulic community with our achieved experimental results and to suggest a possible direction of further research. We assume that further detailed research of fast flow in smooth capillaries below 0.1 mm may bring very interesting applications in the field of “microjet” and in the field of high-velocity fluidics.
